# Supplementary material for: Are carbon emissions trading and green financial instruments synergistic? -Comprehensive quantitative research based on content analysis
Source: PLoS One. 2024 Mar 7;19(3):e0298601. doi: 10.1371/journal.pone.0298601 (PMC10919601; doi:10.1371/journal.pone.0298601)
Supplement: S1 Data — (ZIP) [file pone.0298601.s001.zip › Data/地级市Z_F工作报告.zip!/╣π╬≈/╟╒╓▌╩╨/└┤╫╘╣1⁄2╓┌║┼í╛┬φ┐╦╩2╛▌═°í┐_2015.docx]

各位代表：

　　现在，我代表市人民政府向大会报告工作，请予审议，并请市政协委员和列席会议的同志提出意见。

　　2014年工作回顾

　　过去的一年，是我市社会和谐稳定、经济较快增长的一年。全市认真贯彻自治区党委、政府决策部署，在市委领导下，在市人大、政协监督支持下，深入实施“建大港、兴产业、造新城、强科教、惠民生”发展方略，坚持“稳中求进、提速提质、改革创新、彰显成效”总基调，扎实推进“三个年”活动，沉着应对错综复杂经济形势的挑战，经受住两次超强台风暴雨灾害的考验，经济社会发展迈出了坚实的步伐。

　　——主要经济指标增速排全区前列。初步统计，全市生产总值增长9.8%，居全区第3位，总量855亿元；规模以上工业总产值增长13.9%，居全区第4位，总产值1291亿元；固定资产投资增长17.9%，居全区第4位，投资总额659亿元；外贸进出口增长51.1%，居全区第1位，总额突破50亿美元；社会消费品零售总额增长12.8%，居全区第3位，总额突破300亿元；集装箱吞吐量增长16.8%，达70.2万标箱，居北部湾沿海港口首位。

　　——改革开放内生动力明显增强。重大项目布局速度加快，保税港区、中马钦州产业园区、高新区等重大平台正成为新的增长点。中国－东盟港口城市合作网络钦州基地启动建设。统筹抓好42项重点改革，积极申报建设北部湾自由贸易试验区，强化行政审批制度改革，经济社会发展环境进一步优化。

　　——人民生活水平得到新提高。扎实推进为民办实事工程，解决了一批涉及群众切身利益的医食住行等问题，财政全年用于民生的支出占财政支出比重达到81.4%。城镇登记失业率3.43%，城镇居民人均可支配收入增长7.3%，农民人均纯收入增长10.4%。

　　　　一年来，我们主要抓了六个方面工作：

　　一、坚持以招商引资为抓手，以项目建设为基础，以服务企业为纽带，努力为经济社会发展积蓄力量、释放活力。组织开展“项目推进和招商引资攻坚年”活动，强化重大项目建设指挥部制、市领导联系重大项目责任制，市主要领导定期听取和研究招商工作，继续实施招商引资责任制，形成投资和项目建设新高潮。引进中石油芳烃、修正药业等重大项目，中石油芳烃成功列入全国石化产业规划布局方案，国投电厂二期获核准并全面建设。全年新引进项目369个，其中亿元项目129个，实际到位资金511.7亿元。组织开展“企业服务年”活动，坚持每月开展“市长企业接待日”活动，全年接待企业40家、解决问题98个，落实企业服务联系卡制度，发放联系卡788份，协调办理问题197个，有效帮助企业解决了生产经营和项目建设中遇到的困难。中石油含硫原油加工配套工程全面建成投产，中船修造船一期、30万吨级支航道等重点项目开工，金桂纸业、中粮油脂等企业顺产达产，新投产澄星磷化工等20家企业，化工、浆纸、粮油加工等支柱行业较快增长。科技创新成效凸显，发明专利申请量实现倍增。14个工业技术创新项目列入自治区创新计划，3个创新平台获自治区认定。新增县域亿元企业51家，新增规模以上工业企业12家，县域规模以上工业总产值增长33.4%。出台实施促进非公有制经济发展系列政策，民间投资增长26.1%，非公有制企业注册数量增长20.5%。积极培育财源新增长点，全市财政收入138.3亿元，其中一般公共预算收入47.6亿元，增长6%。

　　二、坚持产城融合发展理念，深入开展“城市建设年”活动，同步推进新城开发、旧城改造和县镇建设，城乡面貌展现新变化。滨海新城、河东新城加快开发，白石湖片区路网等基础设施建设全面推进，北部湾大学等功能性项目稳步建设。实施城建项目195个，完成投资47.8亿元。打通一批断头路，建成一批小街小巷惠民工程，“出行难”得到改善。统筹推进城市环境综合整治，河东、大榄坪等污水处理厂配套管网进一步完善，城市防洪排涝治理初显成效。旧城改造步伐加大，县城及小城镇建设有序开展，城镇基础设施进一步向农村延伸。开展“营销钦州”系列活动，申报建设国家第三批“智慧城市”试点，创“三城”取得阶段性成果。实施“园林生活十年计划”，完成中山公园改扩建、林湖森林公园二期。全市城镇化率进一步提高。

　　三、坚持以开放倒逼改革，港口建设、平台发展和园区开发在扩大开放合作中实现新突破。抓住建设“21世纪海上丝绸之路”和打造中国－东盟合作升级版的机遇，全力推进港口和各类园区的开放开发与合作，外向型经济快速成长。开工建设鹰岭作业区3#、4#泊位等项目，建成永鑫10万吨级散杂货码头、大榄坪南作业区北1－3#泊位等，港口吞吐能力突破1亿吨，吞吐量完成6413万吨，增长6.2%。加密至香港定期航班，成功开通首条至东盟直航航线。中马钦州产业园区获得国务院的特殊政策支持，明确在产业发展、金融创新、土地开发利用等方面先行先试，成为全国外商投资企业资本金结汇管理方式改革试点的16个园区之一；保税港区体制改革获得重大突破，保税港区即将委托钦州管理，二三期通过验收，全面封关运营，获准开展小批量汽车进口，成为进境粮食指定口岸；完成北部湾自由贸易试验区专题研究，总体方案正式上报国务院。率先推进跨境人民币贷款业务试点并开展了广西首笔业务。市直国企改革有序开展。润港林业上报全国中小企业股份转让系统，盛和电子、还珠科技、泰盛木业3家公司在区域性股权市场挂牌。成立北部湾华侨投资区，搭建新的开放平台。与关丹港、西哈努克港结为国际姊妹港，与班达伯格市缔结友好合作城市。进一步调整理顺城区管理机制，积极探索乡镇机构“四合一”改革，政务服务进一步向基层延伸。

　　四、坚持调整产业结构、优化发展空间的思路，推动特色农业提质增效、服务业提速扩量，着力转变经济发展方式。在大力发展二产的同时，加快发展一产和三产，三次产业比例为22.7:39.6:37.7，结构进一步优化。实施“特色农业提升工程”，全力应对超强台风“威马逊”和“海鸥”所带来的超强暴风雨，迅速组织抗灾救灾和灾后恢复生产，粮食、经济作物、畜牧等产量及效益均有增长，水产养殖得到较好恢复；高丰火龙果基地建成首批自治区级现代特色农业（核心）示范区，中国“龟谷”核心区全面开工建设，黄瓜皮、青蟹、红椎菌通过国家地理标志产品保护认证，灵山县荣获“中国茶叶十大转型升级示范县”；农村土地承包经营权确权登记颁证试点稳步推进，新增农民专业合作社246家、家庭农场189家。东盟商贸城一期、东盟农产品大市场一期等专业市场建成，广西南菜北运产地集配中心项目完工。获批筹建4家小额贷款公司和2家融资性担保公司，新引进证券、保险机构各3家。开展全区首笔8亿元保险债权资金业务，成功发行滨海置业集团棚户区改造、临海公司一期企业债券和全区首支8亿元小微企业扶持债券，金融机构本外币存、贷款余额比年初分别增长9%、3%。旅游业进一步发展，三娘湾、八寨沟、五皇山等特色景区改造升级工程建成，千年古陶城加快建设，接待游客和旅游收入分别增长11.5%、22.3%。

　　五、坚持执政为民发展理念，切实加强民生工程、社会事业和生态建设，努力让人民群众得到更多实惠。全力实施为民办十件实事工程，63个子项目完成投资42.8亿元，占计划投资105.6%。建成城区应急水源项目。就业规模持续扩大，保障性安居工程任务超额完成。推进市中医医院等公立医院搬迁新建，建立城镇居民大病救助医疗保险制度，城乡医疗保险实现全覆盖，社会保障体系得到完善。城市和农村低保补助月均分别提高55元、26元，新农合和城镇居民医保政府补助标准提高40元。村（社区）干部待遇月均提高350元。城市公共交通进一步改善，拆迁安置加快推进。建成一批镇村道路，行政村水泥路通畅率达98%，农村群众出行条件大为改善。加强高等教育、职业教育，加快中小学标准化建设，新建和改扩建一批中小学、农村幼儿园，完成老年大学改建项目。建立集聚人才体制机制，引进一批领军型高层次人才。扎实开展文化惠民活动，全面铺开全民健身，成功承办广西第十三届少数民族传统体育运动会。跳岭头列入第四批国家级非物质文化遗产代表性项目名录。扫黄打非工作经验得到国家肯定并在全区推广。优生优育和计划生育服务不断优化，低生育水平保持稳定。继续深化“携手共创平安和谐家园”活动，社会治安持续好转，社会更加和谐稳定。食品药品安全监管不断强化，防震减灾工作获全国先进，应急管理体系进一步完善，安全生产形势保持良好。“美丽钦州·清洁乡村”活动富有成效，农村保洁长效机制逐步构建，生态乡村活动全面启动。强化节能减排，加强钦江流域和茅尾海综合治理，主要污染物总量排放完成自治区下达任务，万元gdp能耗实现下降。工会、共青团、妇联、残疾人事业取得新进展，国防教育和后备力量建设深入开展，双拥共建工作取得新成效，海关、检验检疫、海事、边检、消防、税务、人民银行、银监、工商、质监、供电、烟草、通信、邮政、盐务等为我市经济社会发展作出了新贡献，民族、宗教、外事、侨务、农机、水库移民、供销、广电、统计、贸促、人防、海防、科普、气象、水文、档案、决策咨询、扶贫、地方志、老龄和关心下一代等工作取得新成绩。

　　六、坚持民主决策和依法行政，认真践行群众路线新要求，在反对“四风”立行立改中提升政府形象。深入开展党的群众路线教育实践活动，转变作风，狠抓整改，解决了一批群众反映强烈的突出问题。对政府性楼堂馆所、机构编制进行清理和规范，完善改进作风、加强效能建设、联系服务群众等制度，查处了一批有影响的违纪违法案件，全市“三公”经费下降14%。深入实施行政效能提速工程，进一步简政放权，取消、下放一批行政审批项目，市本级行政审批事项比上年减少14%，投资环境满意度排名全区前列。推进民主决策，自觉接受人大的法律监督、工作监督和政协的民主监督，建立人大代表、政协委员列席市政府常务会议制度，人大代表议案建议和政协提案按时办复率100%。进一步密切与各民主党派、工商联、无党派人士、人民团体和社会各界人士的联系。加强规范性文件、重大行政决策合法性审查。强化行政监察和审计监督，主动公开预决算、环境保护、社会民生等政务信息。

　　各位代表，过去的一年，全市上下从实际出发，务实求进、凝心聚力，担当负重前行，风雨同舟共济，经受住了严峻考验，在面对国际问题与国内矛盾双叠加、短期问题与长期矛盾齐碰头、总量问题与结构矛盾相交织、负面因素不断恶化、整体增速持续放缓、困难矛盾超乎想象的情况下，经济社会发展基础得到有力夯实，取得这样的成绩实在来之不易。这得益于自治区党委、政府和市委的正确领导、科学决策，得益于人大、政协有效监督，得益于法院、检察院的大力支持，得益于各民主党派、工商联、人民团体和无党派人士肝胆相照、携手合作，得益于广大客商和企业家共克时艰、共创大业，得益于驻钦部队、武警官兵、公安干警牢记使命、忠诚履职，也得益于老干部、老同志的理解和支持，得益于全市人民同心同德、艰苦奋斗，大家都是值得点赞的。在此，我代表市人民政府，向大家致以崇高的敬意和衷心的感谢！

　　同时，我们也清醒看到，钦州正处于发展的特殊时期，还存在不少困难和问题。产业结构不优，企业竞争力不强，部分企业生产经营困难；新增长点不多，抗风险能力弱，可用财力不足，偿债进入高峰期；促进民间投资的门槛和障碍依然存在，改革开放内生动力不足，科技创新意识不强、支撑能力不足；资源和环境约束加剧，社会矛盾在短期内难以化解，要素保障和社会稳定压力加大；政府自身建设存在薄弱环节，投资环境有待进一步优化。

　　各位代表，实践证明，困难再大，挑战再多，只要我们勇于担当、砥砺奋进，只要我们坚定不移贯彻中央、自治区决策部署，只要我们从钦州实际出发，保持定力，把牢经济规律，精准发力，坚定不移依靠群众、群策群力，蹄疾步稳加以化解，钦州就一定能再攀高峰、再创辉煌，让钦州人民过上更好的日子！

　　2015年工作安排

　　2015年是全面推进法治建设的开局之年，是“十二五”规划的收官之年。世界经济形势依然复杂严峻，国内经济发展进入新常态，经济下行压力很大。但我市经济发展长期向好的趋势没有改变，特别是国家推进“一带一路”战略和打造中国—东盟合作升级版，自治区全面实施“双核驱动”战略、加快构建新的战略支点，以及国家深化改革、扩大开放所形成的体制机制优势，将为我市加快发展提供更大动力。当前，我市进入工业化和城镇化关键时期，改革开放红利进一步释放，产业发展基础加速形成，我们务必认识新常态、形成新共识，适应新常态、培养新引擎，探索新方式、拓展新空间，以勇立潮头、舍我其谁的气魄，锲而不舍、昂扬向上的精神，只争朝夕、敢为人先的闯劲，推动经济社会发展再上新台阶。

　　今年政府工作的总体要求是：深入贯彻党的十八大、十八届三中、四中全会和习近平总书记系列重要讲话精神，以邓小平理论、“三个代表”重要思想、科学发展观为指导，坚持“稳中求进、扩量提质、改革创新”的工作总基调，坚持以提高经济发展质量和效益为中心，主动适应经济发展新常态，紧紧围绕提前全面建成小康社会目标，全面深化改革、扩大开放，继续实施“三个年”活动，全面推进钦州法治建设，切实保障和改善民生，促进经济平稳健康发展与社会和谐稳定。

　　经济社会发展的主要预期目标是：地区生产总值增长10%；财政收入增长6%；规模以上工业总产值增长18%；固定资产投资增长18%；社会消费品零售总额增长13%；外贸进出口总额增长12%；港口吞吐量7000万吨，其中集装箱吞吐量90万标箱；城镇居民人均可支配收入增长10%，农民人均纯收入增长13%；城镇登记失业率控制在4.5%以内；居民消费价格指数控制在3.5%以内；人口自然增长率控制在10‰以内。

　　围绕目标要求，重点抓好以下六个方面的工作：

　　一、强化项目拉动作用，加快形成经济增长内生机制，培育城市长远发展后劲。

　　大力推进重大项目建设。继续开展“项目推进和招商引资攻坚年”活动。集中攻坚突破中石油芳烃、华谊煤基多联产、汽车、飞机制造、中电投热电联供一期、南防高速公路钦州段改扩建、南宁至北流高速公路钦州段等一批重大项目前期工作，并争取开工建设，形成投资增长新的支撑点。加强协调服务，确保国投电厂二期、中船修造船一期、贵合高速公路等重大项目顺利建设。开工金鼓江12－17#泊位等项目，力争建成30万吨级支航道、金鼓江航道、国投煤炭码头等项目，抓好20万吨集装箱码头及航道前期工作。突出抓好亿元项目储备建设，确保投资平稳和可持续。强化重大项目策划，争取中石油二期、国投电厂三期、中船修造船二期等一批关系钦州长远发展的战略项目，纳入国家和自治区“十三五”规划。

　　坚定不移推进大招商招大商。健全招商引资机制，强化小分队招商、专业招商、产业招商、以商招商和全员招商，保持“啃硬骨头”的攻坚态势和紧叮不放的“蚂蝗”精神。坚持产业龙头和产业链并重的大招商，主要围绕芳烃、煤化工、造纸、汽车、船舶修造五大产业链和高新产业招商，争取一批大项目落户。积极谋划高端装备制造、新材料、新能源汽车等战略性新兴产业，力争有新突破。更加注重引进亿元以上和具有“总部经济”效益的项目，提升招商质效。

　　着力增强港口辐射带动能力。完成5亿吨大港规划、樟木环－观音堂作业区规划调整，加快三墩二期扩区规划研究。全面深度与北港集团合作，推进其与新加坡国际港务、太平船务合作，加快大榄坪片区50平方公里整体开发建设，实现保税港区网内外联动，尽快启动火车东站经中马钦州产业园区至三墩铁路和钦州集装箱办理站的建设，支持北港集团培育以钦州港为中心的集装箱干线港，强化海铁联运，开通“五定”班列，加密至香港班轮，培育开通至东盟外贸集装箱航线，构建与贵阳等腹地城市的“无水港”，确立集装箱干线港的地位。新增中粮码头、大榄坪12－13#泊位等一批涉外码头开放，港口吞吐能力达到1.2亿吨。积极推动平陆运河研究。推进大型汽车站场、火车站场建设，建成运营大榄坪至保税港区铁路支线。开展面向东盟的区域性国际航运中心规划研究和船舶保税登记可行性研究。启动航运服务集聚区规划建设，建成口岸联检大楼、电子口岸及鹰岭、勒沟等作业区围网升级改造工程。在保税港区借鉴实施上海自由贸易试验区监管创新制度。全面实施促进口岸“大通关”的政策措施，探索推进与西南中南跨区域通关合作模式，推动中马“两国一检”查验新模式，推进“六市一关”、“三个一”、“单一窗口”等便利化措施。

　　加强农村基础设施建设。大力推进农田水利建设，开工建设犁头嘴海堤、青年水闸除险重建等项目，加快建设大环及大灶江堤围、石梯水库等项目，完成一批中央财政小型农田水利重点县、病险水库除险加固、海河堤防、中小河流治理、农村饮水安全等项目建设。着力推进119个贫困村精准扶贫，谋划新一轮301个“整村推进”贫困村扶贫攻坚工作，启动76个水库移民新村建设。推进农产品深加工，完善产品加工、冷链物流、销售网点等设施。加大农村生态能源工程和农村路网建设，力争50户以上自然村水泥路通畅率达57%以上。

　　提高发展要素保障水平。全面推进滨海新城、茅尾海综合整治和土地（海域）收储，下功夫解决好农民回建房安置工作。用足城乡建设用地增减挂钩试点和浦北县、钦南区低丘缓坡试点以及钦北区小微企业试点建设用地报批政策。盘活存量土地，着力提高供地率，促进土地集约节约利用。做好耕地“占优补优”，探索实施土地和建设网格化管理，坚决打击违法抢种、抢建行为。扩大资本项目意愿结汇、跨境贸易人民币结算、境外人民币贷款等业务。拓展项目融资渠道，研究建立政府特许经营权管理办法及特许经营体系，统筹各类资金，充分发挥财政资金作用，优先保障在建项目的续建和收尾，重点防范债务风险，积极探索ppp等新的融资方式。

　　二、突出科技支撑作用，促进产业联动发展，加快总量扩张和转型升级。

　　　　壮大提升临港产业。更大力度开展“企业服务年”活动，继续实施“市长企业接待日”制度。加快交通运输业、现代物流业、金融服务业、信息服务业和商务服务业发展。服务好中石油、国投、中粮、金桂等一批骨干企业顺产稳产。支持东油沥青技改、力顺机械转型升级，开工建设东南纸业、富控电梯等项目，加快建设嘉华钛白粉等项目，建成中船修造船一期工程，全面投产中马港青粮油、泰兴石化等项目。规划建设芳烃下游产业园、纺织产业园、纸制品产业园、船舶修造配套区、三墩“石化岛”，形成新的临港产业集聚区。

　　培育做强县域工业。继续实施“县域工业壮大工程”，完善提升县区工业园，打造县域特色工业集聚区。引导有条件的县域工业园区申报自治区级经济技术开发区。建成投产骄丰化工、华益新能源、华汇镍业一期、卓能新能源等项目。继续实施“抓大壮小扶微”工程，支持中小企业发展壮大，创新中小企业融资平台服务，开展“惠企贷”，重点培育发展11个特色产业板块，加快形成一批中小微企业集聚区。推进北部湾华侨投资区开发。力争2015年县域工业产值突破800亿元。

　　提档升级现代服务业。围绕千亿元现代服务业目标，编制现代服务业集聚区发展规划，制定出台扶持政策，促进现代服务业集聚壮大。加快河东新城、滨海新城等商业网点布局，规划建设火车东站商业区，培育金海湾东大街家居建材、小商品专业市场集聚区，建设港航物流、商贸会展、健康医疗、教育科技等服务业集聚区。争取北部湾国际汽车城、红星美凯龙、人和春天商业综合体等大型商业项目开工，推进城区大中型农贸市场和重点镇农贸市场的升级改造。支持和引导电子商务等新型商业业态加快发展。扩大城乡消费市场，充分发挥消费需求的拉动作用。推动特色旅游发展，开通至东盟国家旅游班轮，加快邮轮母港规划研究。开工建设三娘湾文化旅游综合开发、八寨沟旅游综合开发等项目，建成麻蓝岛旅游度假区欢乐海岸、千年古陶城一期等项目，规划建设岭南民俗文化街区，建设国家级非物质文化遗产项目钦州跳岭头传承基地，打造一批文化产品、体育赛事知名品牌，挖掘海洋文化、陶艺文化，培育文化创意、网络服务、数字动漫等新兴文化业态。

　　加快发展特色优势农业。发展现代高效农业，推进农业现代化，实现农业增效、农民增收。培育创建农产品品牌，发展“一镇一业”、“一村一品”。推广应用先进技术，深入实施“米袋子”、“菜篮子”升级工程，建设农产品质量安全检测中心。抓好荔枝、香蕉、大蚝、奶水牛等产业规模化标准化，建设一批标准果园、示范养殖基地。改造提升传统农业，打造一批新型农业示范区，推进北部湾华侨投资区特色农业种植园开发建设，加快火龙果现代特色农业（核心）示范区、台湾农民创业园开发建设，基本建成中国“龟谷”养殖核心区。推进“双高”糖料蔗基地建设。培育一批市级以上龙头企业，支持农民专业合作社示范社和家庭农场建设，新培育农民专业合作社30个。建设玛氏火龙果综合加工基地、龙门港水产产业园等重点农业加工流通项目，力争农产品加工率达到43%以上。

　　大力实施科技创新驱动战略。科技是钦州发展的第一要素，深入实施发明专利倍增计划，启动建设国家级科技孵化研发基地，促进科技创新成果专利化、产业化，引导和支持企业突破一批核心技术，提高企业自主创新能力和科技核心竞争力。加快推进高新区二期建设，围绕2017年创建国家级高新区的目标，重点打造电子、光电、医药、汽车、机电、物流、服务产业园等“园中园”。开工建设富仕通（钦州）产业园项目，争取引进修正集团制药项目，建成投产骏升科技二期、半宙天龙等，促进盛和电子、奥佳华环保电池等稳产达产。利用中马“两国双园”政策优势，扩大引进国内外高新技术要素资源，争取引进北斗东盟产业园等项目，建成投产慧宝源项目。

　　三、实施新型城镇化战略，协调推进中心城区和县镇建设，加快构建新型城镇化体系。

　　推进滨海新城建设上新台阶。继续开展“城市建设年”活动。围绕打造“最美内海城市”，加快白石湖、辣椒槌、沙井岛、茶山江片区开发建设。建成白石湖片区2.3平方公里基础设施、东盟生态园、北部湾国际人才创业基地、风情酒吧街等重点工程。开工建设广西安全工程职业技术学院，加快英华职业技术学院搬迁，开工中央景观大道、奥博体育文化中心、北部湾滨海养老中心、国际海员俱乐部等项目，加快推进北部湾博物馆、沙井岛东岸、九鸦和茶山江安置区、疍家风情小镇等项目建设，引进修正健康产业园等项目。加快推进与中交集团天津航道局合作开发沙井片区。以滨海新城建设为龙头，协调推进河西老城区、河东高新城、港口新城和三娘湾旅游度假新城建设，加快一批功能性项目布局，进一步提升城市对港口和产业的支撑作用。

　　加快完善城市配套功能。坚持规划引领，提升城市综合交通、停车场、给排水、综合管网等专项规划。完善城市路网，加快建设环城东路、环城西路南段、平山东大街、滨海大道以及钦江五桥，开工建设南北二级公路钦州段维修工程、钦江一桥维修加固工程，力争完成环城北路、子材东大街等工程。进一步完善污水垃圾处理设施，重点抓好河东污水处理厂配套管网和生活垃圾收运系统工程建设，建成生活垃圾焚烧发电项目。开展人民路、中山路等历史街区保护规划，实施一马路及周边区域改造，推进上沙村、沙坪村城中村改造，改善老城区交通体系和市政设施，建设城市新型社区。

　　促进产城融合发展。按照产业集聚、项目集中、土地集约、生态环保的原则，加快形成园城协调互动发展格局。高标准规划建设好中马钦州产业园区、保税港区、高新区等一批重点产业园区，打造产业合作示范区。加强县区工业园区和乡镇工业集中区建设，突出抓好钦南进口资源和新材料产业园、钦北皇马工业园建设，推动灵山工业区、浦北县城工业区等自治区产城互动试点园区发展，加快园区路网及生活配套设施建设，以产城一体促进新型工业化与新型城镇化互动发展。

　　统筹兼顾县镇建设。围绕县城和重点镇扩容提质，编制实施新型城镇化发展规划及实施方案。推动灵山、浦北、钦北新区开发建设。规划建设陆屋、寨圩、那丽、大寺、犀牛脚等一批综合型、旅游服务型、工业交通型特色村镇，力争完成全部镇总规及重点镇控规。加快村镇基础设施建设，推进重点镇的路网、给排水、文体设施、绿化、污水及垃圾处理设施建设。推进天然气县县通工程和二级瓶装液化石油气供应站点建设。有序有效开展“镇改办、村改居”工作。

　　着力提升城市管理水平。实行城市精细化管理，提升绿化净化美化水平。加快建设智慧城市，推进城市综合执法体制改革和数字城管建设。深化“三城”创建，深入实施“园林生活十年计划”，抓好黎合江公园、茅尾海红树林生态保护恢复和钦州植物园建设，继续完善林湖森林公园设施，实施城市闲置土地空间“覆绿”工程，加快城市道路绿化精品线路建设。加大城区道路、公园、广场园林绿化和基础设施管护力度，筹备申报第十届广西（钦州）“园博会”。不断完善城市公共交通服务。深化城市环境综合整治，加强城市内涝治理及市政道路维护管理，探索违法建设查控监督考评机制，继续推进钦州湾大道、金海湾大街等市容管理示范街创建。

　　四、坚持深化改革、扩大开放，着力破解经济社会发展的瓶颈制约，切实增强发展动力和活力。

　　深化重点领域改革。开展“改革创新年”活动。深化行政审批制度改革，完成新一轮行政审批项目取消和下放清理工作，继续探索推进乡镇“大部制”改革。加快投融资体制改革，推进工商登记改革，进一步完善配套政策，更大力度支持非公经济发展，推动民营资本进入教育、卫生、医疗、养老、城市基础建设等领域。深化财税体制改革，进一步理顺市和城区、开发区管理体制，探索形成市园良性互动模式。继续研究利用沿边金融综合改革试验区、中马钦州产业园区金融改革的各项政策，探索在股权投资基金、资金池、个人境外投资等领域的改革创新，推进中小企业贷款保证保险试点和上市工作，推动跨境人民币融资业务开展。加快农村体制改革，构建新型农业经营体系，推进土地承包经营权确权登记颁证，建设镇级农村土地流转服务中心和土地流转市场；开展速丰林采伐管理改革试点，推进集体林权配套改革；创新水产畜牧养殖金融服务，继续推进农村信用体系建设，组建农村产权交易中心；积极推进耕地“小块并大块”工作。有序推进户籍制度及相关配套制度改革。落实机关事业单位养老保险制度改革措施，同步完善工资制度。推进医药卫生体制、国企、华侨农林场、公务用车制度等改革。

　　放大开放平台要素集聚和政策效应。健全“市园一体化”的体制和机制，全力支持中马钦州产业园区开发建设，加快形成产业集聚。深化保税港区管理体制改革，探索可持续运营发展模式，将保税港区纳入大榄坪区域统筹开发，加快创建全国首家进口酒类综合服务产业知名品牌示范区，推进国家燕窝及食品营养保健品检测重点实验室建设，力争完成进口肉类、水果、木材口岸报批验收。争取国家尽快批准设立北部湾自由贸易试验区。以中国—东盟海洋合作年为契机，全面建设中国—东盟港口城市合作网络钦州基地。推进钦州港经济技术开发区、整车进口口岸等平台建设，支持各县区开发平台建设，形成开放合力。

　　促进外贸繁荣发展。大力实施加工贸易“三年倍增计划”，大力引进珠三角、港澳台地区转移加工贸易企业，加快培育磷酸出口基地和酒类进口基地，打造加工贸易梯度转移重点承接地。用好外贸政策，争取国家和自治区边境地区转移资金，提升企业发展能力，稳定边境贸易发展。继续加大国际市场开拓力度，全力扩大出口规模。

　　五、大力推进社会建设和民生工程，建立健全生态文明投入机制，切实提升群众幸福指数。

　　突出落实民生保障。继续实施为民办十件实事工程，筹措资金43亿元，实施社保惠民、健康惠民、教育惠民、安居惠民等10类65项重点民生工程。完善社会保险网络和“五险合一”信息管理系统，开展社会保险扩面征缴。开工建设保障性住房和棚户区改造11856套，建成2300套。全面建立“救急难”社会救助制度，在镇（街道）试点设立低收入家庭经济状况核对工作服务站，增强基层社会救助力量。建立基本养老服务制度，建成一批示范中心敬老院，实现30%以上农村社区建有幸福院，改善农村五保供养条件。落实创业奖励政策，开展针对高校毕业生、残疾人、失地农民、失海渔民等群体的特色创业就业技能培训，以培训带动创业就业。加强建设领域农民工工资支付监管，构建和谐劳动关系。

　　健全公共服务体系。继续优先发展教育，推进新一轮学前教育三年行动计划和职业教育攻坚五年计划，建成北部湾大学一期并实现钦州学院整体搬迁，建设和改造一批中小学校及幼儿园，推进教育均衡发展。加快推进市第一人民医院全科医生临床培养基地等项目建设，全面启动新农合市级统筹，提升卫生和计划生育服务水平。支持党建工作站、党群服务中心建设。大力弘扬社会主义核心价值观，加强科普宣传工作，继续实施妇女儿童发展规划。继续推动全民健身，申报坭兴陶世界非物质文化遗产和“海上丝绸之路”世界文化遗产城市。

　　创新社会治理机制。深化“平安钦州”建设，强化社会治安动态管控，注重源头治理，保障人民安居乐业。健全重大决策社会稳定风险评估和应急处置机制。推进社区“网格化”服务管理。依法化解社会矛盾和纠纷，完善信访事项终结评议机制，积极化解信访积案。做好法制宣传教育、法律援助等工作。持续开展安全隐患大检查大整治活动和道路交通安全综合整治行动，着力做好重点行业领域的安全工作，加强人员密集场所事故隐患防范。推进防灾减灾体系基础设施建设，完善灾害预警机制。实施质量兴市战略，推进自治区食品安全城市建设试点工作，全力保障群众舌尖上的安全。

　　推进生态文明建设。发展绿色经济、循环经济和低碳技术，加强节能减排工作，开展钦州港经济技术开发区循环化改造，推进城镇污水处理设施建设及机动车污染减排，完成35家规模化畜禽养殖场污染治理设施建设。强化海洋环境保护工作，重点开展茅尾海综合治理，完善码头污染防治设施，加大对沿海企业的监测监管，加强沿海区域及入海河流流域的环境综合整治，推进监测数据共享，确保海水、江水质量达标。推进造林绿化，加强水土流失综合治理，保护自然生态系统结构和功能。加快可再生能源示范建设。全面推进“美丽钦州•生态乡村”活动，净化美化农村生态环境。

　　六、坚持法治思维、依法施政，善治善政善成，加快建设高效法治政府。

　　更加注重依法行政。加大政务公开力度，自觉接受人大依法监督，主动接受政协民主监督，积极接受舆论监督和社会监督，高度重视司法监督。完善行政权力运行机制。坚持法定职责必须为、法无授权不可为，努力做到政府职权法授、程序法定、行为法限、责任法究。清理规范部门行政权力，推行权力清单制度，完善执法程序，规范执法行为，推进综合执法。推进依法科学民主决策，完善重大行政决策程序，继续邀请人大代表、政协委员列席市政府常务会议。

　　更加注重转变职能。进一步厘清政府与市场、政府与社会以及政府层级之间、政府各部门之间的关系。加快公共资源交易平台建设，稳步推进政府购买服务试点，积极探索公共事务社会化管理。简政放权，加强政府治理能力建设，提高行政效能，坚决杜绝行政不作为、慢作为和乱作为。坚持要理性不要冲动、要排难不要畏难、要担当不要懈怠、要实干不要空谈，形成执政为民、服务发展的良好政风。

　　更加注重从严治政。全面加强惩治和预防腐败体系建设，落实党风廉政建设责任制，强化行政权力的制约和监督，从源头上防治腐败。认真践行“三严三实”，坚决纠正“四风”，厉行节约、反对浪费，从严控制行政成本，坚决制止奢侈浪费。充分发挥审计监督作用，加大纠风和专项治理力度，严肃惩处违法违规行为，解决损害群众利益的突出问题。

　　培育和发展社会组织，支持工会、共青团、妇联等人民团体发挥桥梁纽带作用。加强国防动员、民兵预备役、双拥共建和海防、人防、打私等工作。大力促进民族团结，依法管理宗教事务。继续做好外事、侨务、统计、决策咨询、气象、地震、水文、史志、档案等工作。同时，深入研究和理清新常态大背景下，我市经济社会发展思路，认真谋划好“十三五”经济社会发展蓝图。

　　各位代表！惟其艰难，才更显勇毅；惟其笃行，才弥足珍贵。让我们紧密团结在以习近平同志为总书记的党中央周围，在自治区党委、政府和市委的正确领导下，攻坚克难，锐意进取，为谱写钦州快速发展新篇章、开创更加美好的未来而努力奋斗！
